# Two potent, functionally redundant MazF toxins inactivate the 16S rRNA anti-Shine-Dalgarno sequence and are differentially expressed in *Mycobacterium tuberculosis*

**DOI:** 10.64898/2026.10.23.761202

**Published:** 2026-10-24

**Authors:** Timothy W. Sherrier, Eduardo A. Troian, Unnati Chauhan, Tatsuki Miyamoto, Nancy Woychik

## Abstract

*Mycobacterium tuberculosis* (Mtb) remains a major global health threat, sustained by lengthy, multidrug treatment regimens that promote poor compliance and antibiotic resistance. The pathogen’s ability to evade clearance is facilitated in part by a transition to a nonreplicating, persistent state, a process thought to involve the abundant type II toxin-antitoxin (TA) systems. The Mtb chromosome encodes 11 MazEF TA systems whose MazF toxins are single-strand, sequence-specific endoribonucleases yet the precise RNA targets and physiological consequences of most remain unknown. Here we applied a specialized 5’-end RNA-seq approach to pinpoint the Mtb MazF-mt1 toxin recognition sequence and RNA target. MazF-mt1 specifically cleaves at U↓CCUU within the anti-Shine-Dalgarno (aSD) sequence, releasing it from the 3’ end of the 16S rRNA. Notably, the recently described MazF-mt11 toxin also targets the aSD for removal, cleaving at a different sequence 4 nts upstream of the MazF-mt1 site. Consistent with the essential role of the aSD in translation, both toxins rapidly inhibit new protein synthesis. This functional redundancy prompted us to mine Mtb transcriptomic datasets to compare how the two toxins respond to infection-relevant stresses. MazF-mt1 and MazF-mt11 are differentially regulated following exposure to diverse antibiotic and host-imposed stresses, with distinct temporal expression patterns. Thus, Mtb may coordinate transcription of these functionally redundant toxins to more precisely control the establishment and maintenance of nonreplicating persistence, thereby confounding pathogen clearance.

**Importance:**

The ability of *M. tuberculosis* to resist immune-mediated clearance and antibiotic treatment is central to its success as a pathogen. Here, we identify the target of the endoribonuclease toxin MazF-mt1 as the anti-Shine-Dalgarno sequence near the 3’ end of 16S rRNA, cleavage of which causes rapid growth arrest and translation inhibition. Base-pairing between anti-Shine-Dalgarno/Shine-Dalgarno sequences is required for efficient ribosome recruitment and translation of most bacterial mRNAs. Because we previously reported that the MazF-mt11 toxin also cleaves the 16S rRNA 3’ end to remove the anti-Shine-Dalgarno sequence, MazF-mt1 expression phenocopies MazF-mt11. We further show that this toxin pair responds in contrasting ways to several first- and second-line antibiotics and a wide range of environmental stress conditions. Thus, *M. tuberculosis* encodes functionally redundant, ribosome-targeting toxins that are differentially deployed under stress, illuminating a potential mechanism to rapidly suppress translation and promote entry into a drug-tolerant, nonreplicating persistent state during infection.

